# Applications of Artificial Intelligence in Philadelphia-Negative Myeloproliferative Neoplasms

**DOI:** 10.3390/diagnostics13061123

**Published:** 2023-03-16

**Authors:** Basel Elsayed, Amgad M. Elshoeibi, Mohamed Elhadary, Khaled Ferih, Ahmed Adel Elsabagh, Alaa Rahhal, Mohammad Abu-Tineh, Mohammad S. Afana, Mohammed Abdulgayoom, Mohamed Yassin

**Affiliations:** 1 College of Medicine, QU Health, Qatar University, Doha 2713, Qatar; 2 Pharmacy Department, Heart Hospital, Hamad Medical Corporation (HMC), Doha 3050, Qatar; 3 Hematology Section, Medical Oncology, National Center for Cancer Care and Research (NCCCR), Hamad Medical Corporation (HMC), Doha 3050, Qatar

**Keywords:** artificial intelligence, deep learning, machine learning, convolutional neural networks, clinical decision support system, myeloproliferative neoplasms, diagnosis, prognosis, genomics

## Abstract

Philadelphia-negative (Ph-) myeloproliferative neoplasms (MPNs) are a group of hematopoietic malignancies identified by clonal proliferation of blood cell lineages and encompasses polycythemia vera (PV), essential thrombocythemia (ET), and primary myelofibrosis (PMF). The clinical and laboratory features of Philadelphia-negative MPNs are similar, making them difficult to diagnose, especially in the preliminary stages. Because treatment goals and progression risk differ amongst MPNs, accurate classification and prognostication are critical for optimal management. Artificial intelligence (AI) and machine learning (ML) algorithms provide a plethora of possible tools to clinicians in general, and particularly in the field of malignant hematology, to better improve diagnosis, prognosis, therapy planning, and fundamental knowledge. In this review, we summarize the literature discussing the application of AI and ML algorithms in patients with diagnosed or suspected Philadelphia-negative MPNs. A literature search was conducted on PubMed/MEDLINE, Embase, Scopus, and Web of Science databases and yielded 125 studies, out of which 17 studies were included after screening. The included studies demonstrated the potential for the practical use of ML and AI in the diagnosis, prognosis, and genomic landscaping of patients with Philadelphia-negative MPNs.

## 1. Introduction

Philadelphia chromosome-negative (Ph-) myeloproliferative neoplasms (MPNs) are a group of disorders characterized by acquired mutations within the JAK-STAT signaling pathways of bone marrow stem cells, resulting in the excessive proliferation of red blood cells, white blood cells, or platelets [1,2]. According to the World Health Organization (WHO) classifications, essential thrombocythemia (ET), polycythemia vera (PV), and primary myelofibrosis (PMF) are the three classical Philadelphia-negative MPNs [3,4]. They share clinical and laboratory characteristics that can make them difficult to distinguish, especially in the initial stages of the disease [4,5]. Mutations in one of three genes, JAK2, CALR, and MPL, are found in more than 90% of MPNs, with the remaining 10% of cases identified as triple-negative MPNs [6]. JAK2 mutations, most notably JAK2V617F, are found in almost all cases of PV and more than 60% of ET and PMF, whereas CALR and MPL mutations are found in ET and PMF [7,8,9]. Ph-negative MPNs are associated with high morbidity, reduced quality of life, and decreased overall survival [10]. Accurate categorization of MPNs is essential for effective management since treatment goals and progression risk vary across the diseases [9,11]. Major cardiovascular adverse events and thrombosis are significant contributors to death in patients with Ph-negative MPNs [12,13]. Additionally, patients incur the risk of developing leukemia and fibrosis [14,15].

Artificial intelligence (AI) refers to computer programs that simulate and imitate human intellect, including learning and problem-solving [16]. Machine learning is a branch of artificial intelligence that involves automatically identifying patterns in data [17]. As opposed to having the behavior explicitly written, AI learns to do a task automatically from experience (i.e., data) [18]. Since the turn of the century, advances in computer power and access to ever-growing data repositories have allowed artificial intelligence (AI) to advance rapidly [19]. AI provides numerous potential tools to doctors in general and especially in the field of hematology to better inform diagnosis, prognosis, therapy planning, and basic knowledge relating to malignant hematology [20]. Moreover, subjective histological assessments are currently a significant component of the categorization scheme crucial to the diagnosis of MPNs and various other human cancers [3,4,7]. Recent advancements in computer image analysis have the potential to revolutionize the conventional morphological evaluation of human tissues and can replace or supplement the categorization systems now used in cancers [21,22].

This review summarizes the current literature on the use of artificial intelligence (AI) and machine learning (ML) algorithms in the diagnosis, prognosis, and genomics of Philadelphia-negative myeloproliferative neoplasms (MPNs). The results section is divided into three sub-sections, each exploring a different aspect of MPN research. Section 3.1 discusses the use of AI and ML in MPN diagnosis, including detection, classification, and differentiation between MPN subtypes as well as the role of genomics. Section 3.2 examines the potential of AI and ML in predicting disease outcomes, such as myelofibrosis progression risk and thrombosis, as well as response to therapy using clinical criteria and genomics. This review aims to present an overview of the outcomes, limitations, and future research needs for each of the proposed models.

## 2. Methods

**Figure 1:**
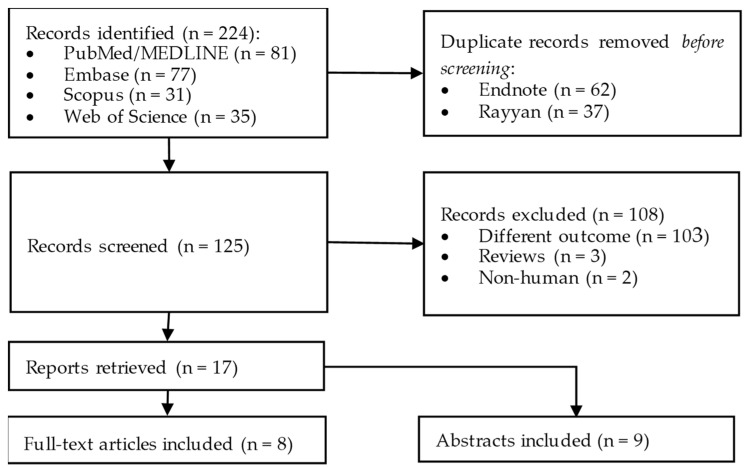
Schematic representation of the literature review process.

Our search strategy (Figure 1) was developed in PubMed/MEDLINE using title/abstract keywords. For Philadelphia-negative MPNs, we included terms such as “myeloproliferative”, “Polycythemia”, “Myelofibrosis”, and “Thrombocythemia”. To review the use of AI in MPNs, we also included terms such as “AI”, “deep-learning”, and “machine-learning”. The combined search was as follows: (“polycythemia”[tiab] OR “myelofibrosis”[tiab] OR “thrombocythemia”[tiab] OR “myeloproliferative”[tiab]) AND (“AI”[tiab] OR “machine learning”[tiab] OR “deep learning”[tiab]). The search was not restricted by language or timeframe. The Polyglot translator was used to convert the initial search strategy to Embase, Web of Science, and Scopus [23]. All the studies identified were moved into EndNote 20 and, subsequently, Rayyan to remove any duplicates [24,25].

This review included research articles that discuss the use of AI and ML algorithms in Philadelphia-negative MPNs in humans. Studies were excluded from our study if they had the following attributes: (1) animal studies, (2) reviews, and/or (3) different outcomes.

## 3. Results

Eight full-text articles and nine conference abstracts were included in our study. Studies were summarized and categorized under two categories: Diagnosis and Prognosis. A summary of outcomes, advantages, disadvantages, AI/ML models and their uses for the included full-text articles can be viewed in Table 1.

### 3.1. Diagnosis

Section 3.1 will focus on the use of AI and ML in the diagnosis of MPNs, including the detection, classification, and differentiation between subtypes of MPNs. This section will explore the potential of these technologies in improving the accuracy and efficiency of MPN diagnosis.

#### 3.1.1. Diagnosis of MPNs Using Bone Marrow and Peripheral Blood Specimens

Sirinukunwattana et al. [26] created a machine-learning method for automated identification, quantitative measurement, and abstract representation of megakaryocyte characteristics utilizing reactive/nonneoplastic bone marrow trephines (BMT) and those from patients with confirmed MPN diagnosis. They discuss the use of an automated method for identifying and delineating key histological characteristics in routinely generated BMTs. Following analysis, tissue diagnosis of MPN was possible with high predictive accuracy (AUC = 0.95), and significant evidence of the ability to distinguish between major MPN subtypes was shown. The machine-learning algorithms described have numerous significant advantages over traditional histology analysis. To begin with, a totally automated model can deliver a rapid and reliable preliminary diagnostic assessment of specimens before official pathology reporting, which is likely to be valuable if access to hematopathology expertise is restricted (for example, in low-resource health care systems). Second, a detailed and interpretable description of the megakaryocytic population will enable the pathologist to concentrate on integrating broader pathological elements with clinical and laboratory data. Third, this approach is well-suited for a more accurate evaluation of consecutive specimens from patients undergoing therapy and/or recurrent examination.

**Figure 2:**
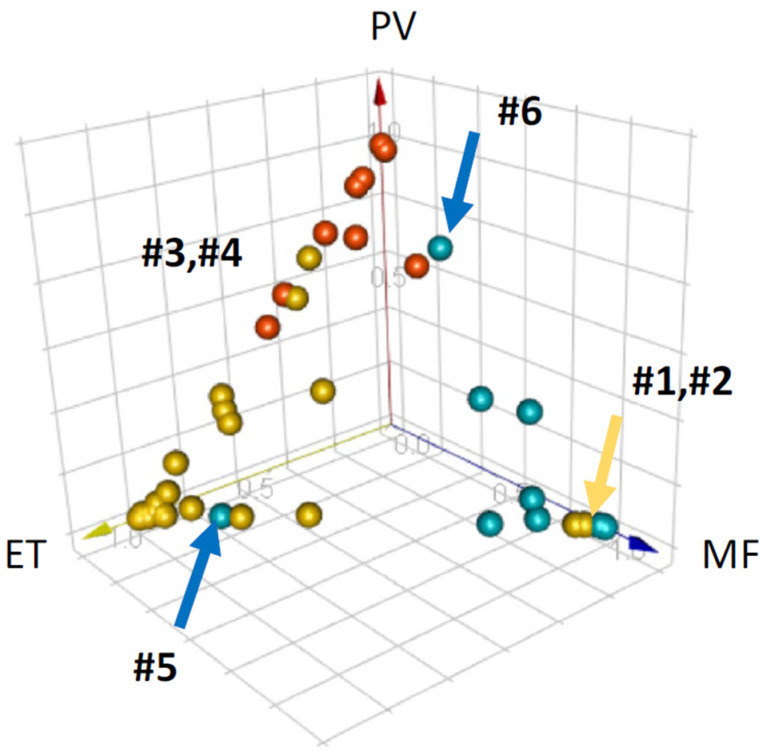
3D plot of the predicted likelihood of Kimura et al.’s automated diagnostic support system with deep learning algorithms for the distinction of myeloproliferative neoplasms. Six misclassified cases are labeled #1–#6.

Kimura et al. [27] established a clinical decision support system for Ph-negative MPNs to minimize workload and inter- and intra-personal discrepancies (Figure 2). The technique involved combining the complete blood counts (CBCs) and research data collected by an automated hematological analyzer (Sysmex XN-9000) with peripheral blood (PB) smear morphological features retrieved using a recently developed Convolutional Neural Network (CNN) coupled with an Extreme Gradient Boosting (XGBoost)-based decision-making algorithm [34,35]. In brief, a deep learning system (DLS) was trained using 695,030 normal and pathological cell pictures, 174 XN-9000 parameters, and 114 cell morphological parameters through a CNN-based image-recognition system. The DLS could successfully categorize 17 cell subtypes and detect 97 aberrant morphological characteristics after training. The diagnostic-aid algorithm was also trained to distinguish between 23 polycythemia vera (PV), 101 essential thrombocytosis (ET), and 36 myelofibrosis (MF) cases. The system was evaluated utilizing samples from 9 PV, 53 ET, and 12 MF patients and demonstrated reliable performance in distinguishing PV, ET, and MF with high accuracy when compared to human diagnoses, specifically with >90% sensitivity and >90% specificity. The computed area under the curve of the ROC curves for PV, ET, and MF, respectively, were 0.990, 0.967, and 0.974. This work adds to previous AI-based studies utilizing mutational data, peripheral blood specimens, and bone marrow trephines for a more accurate diagnosis of MPNs [36,37,38].

#### 3.1.2. Differential Diagnosis of PMF and ET Using Megakaryocytic Lineage

Asaulenko et al. [28] created machine learning algorithms capable of distinguishing morphological and histotopographical aspects of bone marrow samples in order to increase the accuracy of essential thrombocythemia (ET) and prefibrotic primary myelofibrosis (prePMF) differential diagnosis. The Density-Based Spatial Clustering of Applications with Noise (DBSCAN) clustering technique was developed in-house and was used to investigate particular histotopographical aspects of the megakaryocytic lineage in bone marrow samples from 95 patients with JAK2- or CALR-mutated ET and prePMF. The percentage of correct predictions for ET (90%) was much greater than prePMF (40%). The overall correct diagnostic probability was 71.6%. By building logistic regression models with 75% and 25% of the source dataset, the model was verified in a training and testing sample. The ROC was constructed, and the area under the curve was 72.5%. The discrepancies found by the DBSCAN clustering technique suggest that there may be a connection between the MPN subtype and the development of the megakaryocytic lineage. The results of this study agree with a recent study by Yassin et al. [39] assessing bone marrow activity using 3′-18Fluoro-3′-deoxy-L-thymidine (18F-FLT) with positron emission tomography (PET) to differentiate between patients with essential thrombocythemia and prefibrotic primary myelofibrosis. The findings demonstrate that the characteristics of the bone marrow in MPNs differ in a manner that cannot be visually observed during microscopic analysis and can only be detected using machine learning and advanced automated imaging techniques.

#### 3.1.3. Reduced and Optimized PV Diagnosis Rules

In 2002, polycythemia vera was often diagnosed using the widely used Polycythemia Vera Study Group (PVSG) diagnostic criteria [40,41,42]. Kantardzic et al. [29] introduced a data-mining method to derive new decision criteria for polycythemia vera diagnosis based on a reduced and optimized subset of lab characteristics. This study included several laboratory and clinical findings from 431 polycythemia vera patients and 91 patients with other myeloproliferative disorders within the original dataset used in the PVSG study. They demonstrated that it is feasible to reach a diagnostic conclusion about polycythemia vera with the same degree of classification quality while utilizing only four parameters: hematocrit, platelet count, splenomegaly, and white blood cell count, using typical data-mining approaches such as artificial neural networks, support vector machines, and n-dimensional visualization. The categorization of polycythemia vera patients using a trained artificial neural network or a support vector machine compared to PVSG diagnostic criteria, the gold standard for polycythemia vera diagnosis at the time, did not show any significant differences. Therefore, these reduced and optimized diagnostic criteria could be utilized in addition to the conventional PVSG criteria, notably in differentiating polycythemia vera from other myeloproliferative syndromes.

#### 3.1.4. Diagnosis of JAK2 V617F Negative Patients with WHO-Defined ET

Smart Blood Analytics (SBA) is a machine learning-based application that examines complete blood counts (CBCs) to generate a list of probable diagnoses. It is primarily proposed for medical professionals who lack a thorough grasp of hematology and has been proven to be useful in diagnosing hematological illnesses [43]. Belcic et al. [44] further evaluated the SBA algorithm’s applicability in 237 patients referred for suspected essential thrombocythemia (ET). They classified individuals as having ET according to WHO 2008 or modified WHO criteria. All 237 patients were then assessed using the SBA algorithm. The SBA algorithm detected ET patients with 100% sensitivity and 50% specificity. The SBA machine-learning method may be effective in situations where expert consultation is not accessible.

#### 3.1.5. Supervised Classification of MPNs

Skov et al. [45] studied the possibility of categorizing ET, PV, and PMF into independent categories using supervised classification techniques and looked into the possibility of overlap between the three entities. Blood specimens from 19 patients with ET, 18 with PV, and 9 with PMF were used in gene expression microarray investigations. In order to select the best multi-class classification technique, 11 support vector machine-learning algorithms were used in the voting process. Five gene sets were utilized to train the models, with one set left out for cross-validation. The categorization techniques were established to have optimal accuracy and significance level. In summary, 15 patients (79%) with ET, 28 patients (68%) with PV, and 8 patients (89%) with PMF were accurately predicted by the support vector algorithms. The categorization models have a 79% overall accuracy and performed quite well statistically (*p* = 1 × 10^−6^). These results suggested that ET, PV, and PMF might be classified as discrete disease entities, with overlap between ET and PV providing support to a biological continuum from ET through PV to PMF. Furthermore, this supervised multi-classifier might help with the differential diagnosis of MPNs.

#### 3.1.6. Bayesian Networks Elucidate Complex Genomic Landscapes in MPNs

**Figure 3:**
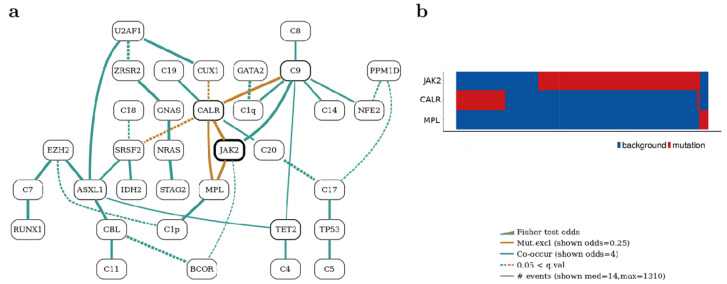
(**a**) Angelopoulos et al.’s Bayesian Network of the myeloproliferative neoplasms dataset; (**b**) Heatmap plots for presence (red) or absence (blue) of driver mutations in CALR, JAK2, and MPL genes on the *y*-axis against patients on the *x*-axis.

Bayesian networks (BNs) are artificial intelligence (AI) models that can define complex joint probability regions. Angelopoulos et al. [32] used the BN model to analyze genetic data from 2035 individuals with myeloproliferative neoplasms and discovered a number of genomic groupings in myeloproliferative neoplasms (Figure 3). For example, mutations in TP53 co-occurred with chromosomes 17p and 5q29 deletions. Another grouping, characterized by LOH at chromosome 4q, abnormalities in chromosomes 7 and 7q occurring in conjunction with mutations in at least 14 myeloid malignancies, accounting for the largest co-mutation pattern in the Bayesian network, was enriched for patients with PMF. Moreover, JAK2 was identified as the most mutated gene in the network, with CALR and MPL mutations exhibiting reciprocal exclusivity patterns, demonstrating functional redundancy in their pathogenic processes. The three genes (JAK2, CARL, and MPL) accounted for most of the dataset’s total driver mutations. The BN model beautifully depicts the major players’ three-way mutual exclusivity. Moreover, the genomic landscapes elucidated by the BNs are supported by clinical exome sequencing studies assessing driver mutations in MPNs [46,47]. Understanding the genomic landscape of MPNs using AI may prove useful in developing diagnostic panels that complement and confirm clinical diagnoses.

#### 3.1.7. Distinction of MPNs Using Genetic Markers

Meggendorfer et al. [48] combined genetic profiling using AI-augmented next-generation sequencing (NGS) with clinical parameters to differentiate and classify MPNs. Firstly, deep-learning algorithms were applied to NGS and allowed the exploration of 15 different genetic markers in addition to JAK2, CALR, and MP. Secondly, clinical parameters were combined with the 18 genes and applied to a training cohort of 243 Ph-negative MPNs using support vector machines (SVM). Individual models for PMF, PV, and ET were created using threefold cross-validation. Then, the trained models were utilized to estimate the most likely diagnosis of patients from a testing cohort, including 183 MPN patients, with the class probabilities for each patient retrieved. Overall, an accuracy of 96% was achieved, demonstrating the potential of deep learning to assist physicians by making the diagnosis based on the patient’s genetic background, even if certain clinical criteria were absent.

#### 3.1.8. Random Forest Classifier for Predicting MPN Subtype Using Genomics

Jabalameli et al. [49] used machine-learning methods to analyze germline genetic variations and built a robust prediction model for ET and PV patients. Using a patient’s genetic data; a Random Forest Classifier was used to predict MPN subtypes. The Illumina Human OmniExpressExome-v1.2 was used to genotype 499 JAK2+ve ET cases and 505 JAK2+ve PV cases. Single nucleotide polymorphisms (SNPs) with a minor allele frequency (MAF) of <0.01, SNPs and samples with more than 10% missing genotypes, and SNPs deviating from Hardy–Weinberg Equilibrium (HWE) proportions were all removed for quality control. SNPs with r^2^ > 0.2 within a 50 kb frame were eliminated to account for multi-collinearity and avoid overfitting. For data visualization and categorization, Principal component analysis (PCA) was employed as an unsupervised technique. To build the classifier model, 7144 SNPs with significant alternative allele relationships (*p* > 0.05) between ET and PV were employed. The MPN ensemble classifier model proved extremely accurate in predicting MPN subtypes. Using fivefold cross-validation, the Random Forest model (AUC = 90%) outperformed the Decision Tree model (AUC = 87%) in terms of accuracy. The findings show that germline variation information may be used to predict the MPN subtype using machine-learning modeling.

### 3.2. Prognosis

Section 3.2 will examine the potential of AI and ML in predicting disease outcomes, such as the risk of myelofibrosis progression and thrombosis, as well as response to therapy. This section will explore the potential of these technologies in guiding personalized treatment decisions for MPN patients.

#### 3.2.1. Myelofibrosis Prediction Using Platelet Transcriptome of MPNs

**Figure 4:**
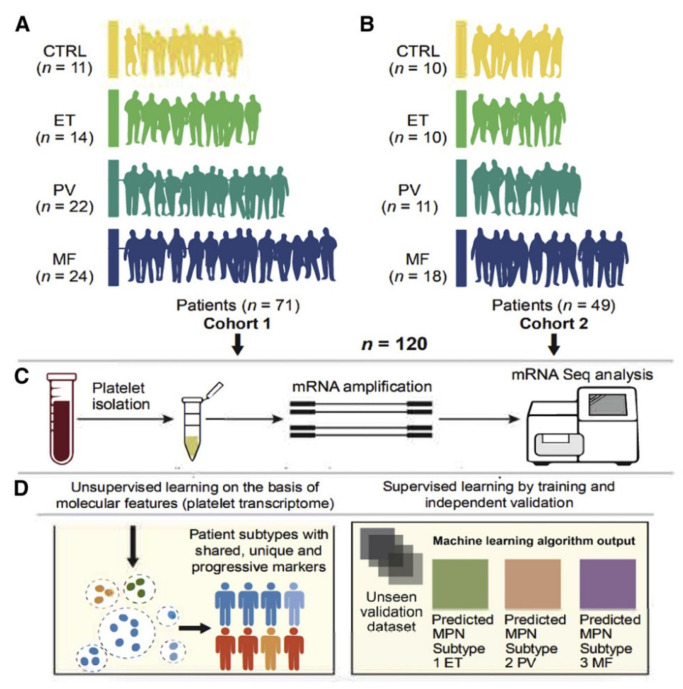
Graphical abstract of Shen et al.’s machine learning algorithm for myelofibrosis prediction using platelet transcriptome of myeloproliferative neoplasms. (**A**,**B**) Two independent cohorts of MPN patients and healthy controls were included. (**C**) Platelets from peripheral blood samples of the two cohorts were isolated and sequenced to yield a platelet transcriptome. (**D**) Machine learning approaches, including unsupervised and supervised techniques, were utilized for myelofibrosis prediction using the platelet transcriptome.

The capacity to anticipate how a patient’s condition will develop or change over time, from essential thrombocythemia (ET) or polycythemia vera (PV) diagnoses to secondary myelofibrosis (SMF), is currently restricted by an understanding of genetic, cytogenetic, or epigenetic variances in myeloproliferative neoplasms. [50,51]. Shen et al. [30] built multiple LASSO (Least Absolute Shrinkage and Selection Operator) penalized regression classifiers [52] that use machine learning to evaluate platelet transcriptome in order to distinguish MPN subtypes and to allow MF prediction (Figure 4). Aside from a training set, two robust external validation criteria [53] were used: temporal validation with two independent cohorts and geographical validation using independently published datasets on healthy donors, and patients with MF [54,55]. The best-performing model employed a collection of progressively differentiated MPN genes and identified five potential genetic markers as the top predictors of disease development. Essentially, LASSO regression and progressive transcriptome studies yielded a compact gene signature that might be used in a PCR-based predictive assessment and performed in clinical laboratories.

#### 3.2.2. Continuous Index of Fibrosis in MPNs

**Figure 5:**
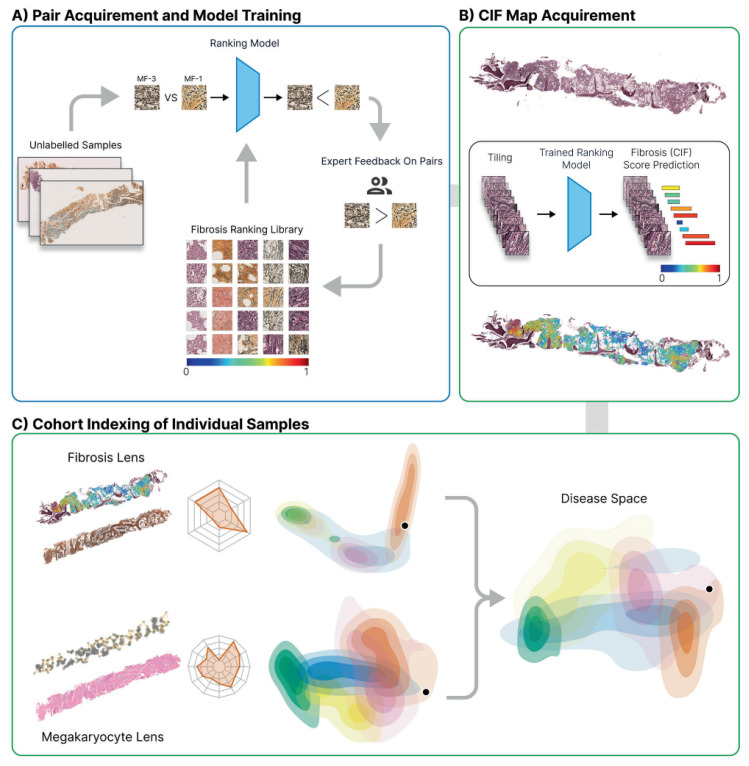
Ryou et al.’s computational steps for detection, quantitation, and visualization of reticulin fibrosis in BMTs. (**A**) Image tiles are labeled with fibrosis grade and used to train a ranking-CNN model that was refined using a human-in-the-loop approach. (**B**) The finalized model generates CIF maps by predicting scores and overlaying them on reticulin-stained images. (**C**) Fibrosis features are represented in a two-dimensional disease space, enabling comparison of MPN subtypes and individual patient BMTs against a sample library.

In MPNs, fibrosis grading is a significant component of disease categorization, prognosis, and surveillance [3]. Ryou et al. [31] devised a machine-learning (ML) approach to objectively measure bone marrow reticulin fibrosis and enhance fibrosis grading (Figure 5). Manually labeled areas of fibrosis were utilized to train the model, yielding a continuous index of fibrosis (CIF) ranging from 0 to 1. The projected scores of new samples were then translated into a quantitative fibrosis map using artificial intelligence, which was subsequently superimposed over entire sample images. The whole spectrum and variety of fibrosis among established MPN and normal/reactive BMT samples are captured in analyses of MPN sample cohorts. Additionally, when paired with megakaryocyte analysis, CIF has good predictive accuracy (AUC = 0.94) in distinguishing between the typically difficult differential diagnosis of ET and pre-fibrotic MF. CIF also showed promise in identifying MPN patients at risk of disease progression. When CIF was tested on a study of 35 ET patients engaged in the Primary Thrombocythemia-1 trial, the model discovered characteristics predictive of post-ET MF with relatively high accuracy (AUC = 0.77) [56].

#### 3.2.3. Predicting Risk of Thrombosis in PV Using Clinicopathologic Features

Using the most significant of 60 clinicopathologic variables, Abu-Zeinah et al. [57] developed a machine-learning algorithm-based clinical decision support system that predicts the incidence of thrombosis in polycythemia vera patients. Prediction of arterial or venous thrombosis in the following 3 months was performed using a random survival forest (RSF) model. A cohort of 470 patients with polycythemia vera was obtained from Weill Cornell Medicine’s database and divided into training (75%) and testing (25%). The grading system was created using the most important ML-derived characteristics and validated using multivariable logistic regression analysis. Using the Fine–Gray model, the cumulative incidence (CI) of thrombosis was compared between risk categories. Five of the top ten variables that independently predicted thrombosis within the RSF model were included in a clinical decision support system that quantified thrombosis risk. A point was allocated for the presence of each of the five variables: age, old thrombosis, recent thrombosis, leukocytosis, and recent diagnosis. The cohort was then labeled as low, intermediate, and high-risk if their score in the grading system was 1/5, 2/5, or ≥3/5, respectively. The grading method revealed that high-risk and intermediate-risk individuals were 6.5 and 2.3 times more likely to develop thrombosis, respectively, than low-risk patients, and the likelihood of thrombosis was substantially different after 1, 2, and 5 years.

#### 3.2.4. Predicting Fibrosis in PV Using Accessible Baseline Characteristics

By using easily accessible baseline parameters, Srisuwananukorn et al. [58] created a machine-learning algorithm that predicts the development of myelofibrosis in polycythemia vera (PV) at the time of diagnosis using characteristics such as demographics, physical exam findings, and laboratory values. A total of 35 models were created after dividing a cohort of 527 PV patients into a development set (90%) and a hold-out set (10%) based on disease progression status. A set of biologically plausible characteristics were chosen using minimum depth analysis across all 35 models. The models were compared to well-known prediction models such as the European LeukemiaNet (ELN) score and the International Working Group MPN Research and Treatment (IWG-MRT) score. Age and past thrombosis define the ELN score, while age, WBC, and prior thrombosis determine the IWG-MRT score. Three variables—age, neutrophilia, and leukocytosis—were utilized as continuous values in a final random survival forest (RSF) model. The model outperformed the ELN and the IWG-MRT scores (*p* = 0.001) and is undergoing development to become an interactive online interface.

#### 3.2.5. Predicting Hydroxyurea Failure and Thromboembolism in PV

Verstovsek et al. [59] developed an accurate and reliable machine-learning model that forecasts the incidence of thromboembolism (TE) in polycythemia vera (PV) patients undergoing Hydroxyurea (HU) treatment. The model was trained using 69,464 polycythemia vera patients within the US OPTUM database. For prediction, a logistic regression model was constructed, and the results were transformed into therapeutically applicable decision-tree classification algorithms. Lymphocytosis and high red cell distribution width (RDW) were predictive for patients without a history of TEs, while thrombocytosis and lymphocytosis were predictors in patients with a history of TEs. Similarly, high RDW was predictive of HU failure in phlebotomy-dependent patients after three months of treatment. Additionally, PV patients who switched to Ruxolitinib had a lower incidence of TEs than those who stayed on HU.

#### 3.2.6. Predicting Thrombosis Risk in Secondary Myelofibrosis

Mora et al. [60] examined thrombosis predictors in the MYSEC PM (Myelofibrosis Secondary to PV and ET-Prognostic Model) database. A machine-learning random survival forest (RSF) model that incorporated phenotypic and genotypic variables upon secondary myelofibrosis (SMF) diagnosis was utilized to find predictors of thrombosis incidence such as gender, age, RBC indices, WBC indices, bone marrow fibrosis, and prior thrombosis. Then, in order to assess the function of the ML algorithm in predicting thrombotic risk, a second model was developed, which aggregates MYSEC-PM components. The results have shown that the random forest model involving MYSEC-PM and past thrombosis predicted thrombotic risk following SMF evolution with great accuracy.

#### 3.2.7. Prediction of Primary Myelofibrosis Using Gene Expression

Li et al. [33] sought to uncover unique diagnostic and predictive gene expression profiles of primary myelofibrosis (PMF) from gene expression datasets. Weighted Gene Co-expression Network Analysis (WGCNA) methods were used to find the most associated genes to PMF. Following that, Gene Ontology (GO) and Kyoto Encyclopedia Genes and Genomes (KEGG) searches were conducted in order to properly comprehend the specific information of a module of interest labeled Green. Following WGCNA analysis, module Green was substantially associated with PMF disease. Twenty genes in module Green were identified as hub genes for PMF development. EPB42, CALR, SLC4A1, and MPL were the most highly expressed and correlated with PMF, and a machine-learning model was developed to demonstrate the reliability of these genes. The SVM (support vector machine) was chosen as the best-suited prediction model and was assessed in third-party datasets, obtaining AUCs of 0.922 and 0.875 within Gene Expression Omnibus (GEO) datasets GSE53482 and GSE61629, respectively.

#### 3.2.8. Prediction of Myelofibrosis Progression in Health Records

Bejan et al. [61] devised a system to predict myelofibrosis using Natural language Processing (NLP) machine learning with negation detection of MF keywords, drugs, and ICD coding to construct phenotype-genotype connections. JAKextractor, an algorithm to find individuals clinically tested for JAK2V617F across 248,000 cases in BioVU, a bank of de-identified DNA samples, was developed to enhance the dataset. A supervised learning system was taught to learn decision rules that encode MF-specific ICD codes, drugs, the text mentions, and the assertion status of MF and JAK2 references in patient notes for MF identification. A 10-fold cross-validation technique was used to evaluate the experiments. JAKextractor employed pattern matching to determine the state of each JAK2 text mention (wild-type versus mutated). Based on the information gathered from patient notes, machine learning identified JAK2V617F patients. The best-performing MF algorithm included all clinical data sources and attained an F1-measure (F1) of 96%, identifying 309 MF patients in BioVU. Moreover, 71/131 (54.2%) of MF patients were genotyped with JAK2V617F, compared to 66/131 (50.4%) identified using JAKextractor. There were only two false positives and four false negatives in the JAKextractor predictions. These findings confirmed the possibility of constructing an MPN database using retrospective genotyping of biobanked DNA and demonstrated the effective identification of MF and JAK2V617F within an electronic health record.

## 4. Discussion

While the use of AI and ML technologies in MPNs has shown promise, several limitations should be considered. Firstly, the use of these technologies may not be diagnostic by itself and only complements the standard diagnostic criteria for MPNs, such as the requirement for marrow cellularity, lineage maturation, degree of fibrosis, and blast cell estimation, which are necessary for MPNs WHO classification. Therefore, the use of AI and ML should not be relied upon solely for diagnosis but rather as a tool to assist physicians in making a diagnosis. Secondly, many of the reviewed studies were based on single-center and a small number of cases, which may limit the generalizability of the results. Additionally, some of the reviewed studies only analyzed cell-derived molecular alterations, indicating that biological and computational validations are necessary for decision-making. Furthermore, the application of AI and ML technologies in MPNs may be based on invasive techniques that require BM trephines, which may not be suitable for all patients and therefore limit the utility of these technologies. Finally, some of the reviewed studies focused on larger datasets and molecular biology, such as the identification of hub genes or the use of Bayesian networks, which may require larger samples to validate the proposed models. Therefore, future studies should focus on addressing these limitations to fully realize the potential of AI and ML technologies in MPNs research and clinical practice.

## 5. Conclusions

This literature review summarizes the recent advances in the applications of Artificial Intelligence (AI) within Philadelphia-negative (Ph-) myeloproliferative neoplasms (MPNs). Machine learning, deep learning and digital hematopathology models have shown promising results in diagnosing, prognosing and explaining the complex genomics of Ph-MPNs with remarkably high accuracy and reliability. As management of the complex and overlapping MPNs depends upon correct classification, AI models might prove useful in the treatment and monitoring of patient suffering from these rare disorders. Nevertheless, in order to assess the effectiveness of these models in diverse clinical scenarios, additional research studies utilizing larger samples and improved methods are still required.

## Figures and Tables

**Table 1:** Summary of included full-text articles.

Study	Outcome	Advantages	Limitations	Model(s)	Model(s) Uses
Sirinukunwattana et al. [26]	Automated analysis of megakaryocytes can categorize MPNs and differentiate them from reactive BM samples	Fast assessment of sequential BM samples Comprehensive summary of megakaryocytic cells	Marrow cellularity, lineage maturation, degree of fibrosis, and blast cell estimation are required for MPNs WHO classification	Unsupervised Learning: Principal Component Analysis (PCA)	Reduction of high dimensional data Exploratory data analysis and visualization of complex datasets
Kimura et al. [27]	Automated diagnostic support system for MPNs using peripheral blood (PB) specimen	Fast assessment of PB specimens Accurate differentiation of PV, ET, and MF	Single-center study Small number of cases	Deep Learning: Convolutional Neural Network (CNN)	Image recognition and classification Learns features automatically from raw data
Asaulenko et al. [28]	Histotopographical features of megakaryocytes allowed correct differentiation between ET and PMF in 71.6% of cases	Patterns of megakaryocyte distribution in BM of ET and prePMF patients with JAK2/CALR mutations can be revealed only using ML	The percentage of correct diagnostic predictions for PMF was only 40%	Unsupervised Learning: Density-Based Spatial Clustering of Applications with Noise (DBSCAN)	Clustering and anomaly detection in high-dimensional data Automatically detect clusters of arbitrary shape and size
Kantardzic et al. [29]	Extraction of new decision rules for PV diagnosis, based on a reduced and optimized set of lab parameters	Reducing the original parameters of diagnosis to only four while still obtaining good classification results	Not diagnostic by its own Only complements the standard PVSG criteria	Supervised Learning: Artificial neural networks (ANNs) and Support vector machines (SVMs)	Classification and regression tasks Both require large amounts of labeled data for training
Shen et al. [30]	Progressive platelet transcriptomic markers, enable an externally validated prediction for advanced MPNs	Comprehensive catalog of platelet transcriptome in chronic MPNs Accurate prediction of MF using <5 candidate markers	Only analyzed platelet-derived molecular alterations Biological and computational validations are needed for decision making	Supervised Learning: Multiple LASSO (Least Absolute Shrinkage and Selection Operator) penalized regression classifiers	Classification and prediction tasks, especially when there are more features than observations
Ryou et al. [31]	Continuous Indexing of Fibrosis (CIF) enhances the detection and monitoring of fibrosis within BMTs and aids MPN subtyping	Accurate discrimination between ET and Pre-PMF Identification of MPN patients at risk of progression	Invasive technique Requires BM trephines	Ranking-CNN: Learning to Rank (LTR) and Convolutional Neural Network (CNN)	Ranking and recommendation tasks LTR models rank items based on relevance, while CNNs extract features from raw data
Angelopoulos et al. [32]	Discovered genomic sets and their relationships in MPNs using Bayesian networks (BNs)	BNs allow correlations among driver events in large genomic cohorts Graphical illustrations	Robustness of the networks is important for BN learning Not suitable for small datasets	Probabilistic Graphical: Bayesian networks	Probabilistic inference and decision-making under uncertainty Models’ complex relationships between variables
Li et al. [33]	EPB42, CALR, SLC4A1 and MPL are candidate prognostic biomarkers and potential therapeutic targets for early PMF	WGCNA, a powerful global research tool for data mining from multiple genes in large-scale datasets was used	Molecular biological studies and larger samples are needed to further validate these hub genes	Supervised Learning: Support vector machines (SVMs)	Classification and prediction tasks, especially when there are more features than observations

## Data Availability

Not applicable.
